# Comparative analysis of transposable elements provides insights into genome evolution in the genus *Camelus*

**DOI:** 10.1186/s12864-021-08117-9

**Published:** 2021-11-20

**Authors:** Mohanad A. Ibrahim, Badr M. Al-Shomrani, Mathew Simenc, Sultan N. Alharbi, Fahad H. Alqahtani, Mohamed B. Al-Fageeh, Manee M. Manee

**Affiliations:** 1 National Center for Bioinformatics, King Abdulaziz City for Science and Technology, Riyadh, Saudi Arabia; 2 Department of Biological Sciences, California State University, Fullerton, USA; 3 Life Sciences and Environment Research Institute, King Abdulaziz City for Science and Technology, Riyadh, Saudi Arabia

**Keywords:** Camelid genomes, Transposable elements, De novo TEs annotation, Transposons, Retrotransposons

## Abstract

**Background:**

Transposable elements (TEs) are common features in eukaryotic genomes that are known to affect genome evolution critically and to play roles in gene regulation. Vertebrate genomes are dominated by TEs, which can reach copy numbers in the hundreds of thousands. To date, details regarding the presence and characteristics of TEs in camelid genomes have not been made available.

**Results:**

We conducted a genome-wide comparative analysis of camelid TEs, focusing on the identification of TEs and elucidation of transposition histories in four species: *Camelus dromedarius*, *C. bactrianus*, *C. ferus*, and *Vicugna pacos*. Our TE library was created using both de novo structure-based and homology-based searching strategies (https://github.com/kacst-bioinfo-lab/TE_ideintification_pipeline). Annotation results indicated a similar proportion of each genomes comprising TEs (35–36%). Class I LTR retrotransposons comprised 16–20% of genomes, and mostly consisted of the endogenous retroviruses (ERVs) groups ERVL, ERVL-MaLR, ERV_classI, and ERV_classII. Non-LTR elements comprised about 12% of genomes and consisted of SINEs (MIRs) and the LINE superfamilies LINE1, LINE2, L3/CR1, and RTE clades. Least represented were the Class II DNA transposons (2%), consisting of hAT-Charlie, TcMar-Tigger, and Helitron elements and comprising about 1–2% of each genome.

**Conclusions:**

The findings of the present study revealed that the distribution of transposable elements across camelid genomes is approximately similar. This investigation presents a characterization of TE content in four camelid to contribute to developing a better understanding of camelid genome architecture and evolution.

## Background

Transposable elements (TEs) are influential in determining genome structural dynamics. TEs are DNA sequences found in nearly all eukaryotes which encode various proteins which carry out the molecular mechanisms which facilitate their relocation and duplication within a host genome [103]. They can comprise substantial proportions of eukaryotic genomes, for example, 50% of the human genome, up to 90% of the maize genome, and varying from 2.7% to 47% of insect genomes [46]. The ability of a TE to proliferate within a genome contributes to it undergoing natural selection as a discrete evolutionary entity, separate from its host. Given this, TEs can be viewed as selfish intragenomic parasitic sequences [27] because their ability to undergo replicative transposition via an RNA or DNA intermediate is not seen as adaptive in most cases. The abundance and repetitive nature of TEs are among the main challenges that complicate the correct assembly of sequenced genomes [96]. However, the significantly repetitive nature of the genomes of mammals and other vertebrates also plays an essential role in various processes, contributing substantially to genome size and architecture [24, 30, 55] as well as influencing functional genomic components [14]. The technological developments in genomics and large-scale functional assays has spotlighted the multi-faceted properties of TEs and their importance in shaping genomes [15].

We are at the early stages of understanding how mobile element insertions influence specific phenotypes. TEs can disrupt host sequences and act as substrates for nonhomologous recombination, forming DNA rearrangements such as deletions, duplications, inversions, and translocations [36, 47]. Such rearrangements can be deleterious for the host through the alteration of gene-coding potential and regulation or by modifying other necessary genomic sequences [58]. TEs are, therefore, causes of mutations and genetic diseases in humans and other organisms [41, 97]. In some cases, TEs are also proposed to be involved in the rapid adaptation of invasive species to new environments [19]. Environmental stressors represent a daily challenge for some organisms, who must adapt to survive continuously changing conditions [20]. An increasing number of studies support a link between TE activity and species responsiveness to environmental conditions [35, 64]. In this context, mobile elements contribute to increasing genetic diversity, allowing organisms to better adapt to new conditions [91]. Given this, TE activity may have contributed to the high diversity of vertebrate species that colonized many habitats, from water to land and temperate to extreme environments [19].

Here, we have analyzed the global TE content in four camelid genomes, contributing to a better understanding of the genome organization and evolution in camelids. *Camelus dromedarius*, frequently referred to as the Arabian camel, is a heat stress-resistant animal [67] capable of living in the extremely harsh climates of the Arabian Peninsula. The adaptations of camelids to arid conditions are remarkable. Camels can fluctuate their body temperature from 34 ^∘^ C to 41.7 ^∘^ C and can conserve water by not sweating [2]. Additional members of the camelid family included in our study are the Bactrian camel (*Camelus bactrianus*) and the Wild Bactrian camel (*Camelus ferus*) of Asia, and the alpaca (*Vicugna pacos*) of South America [4, 38]. The extreme variation among their natural habitats opens up a series of questions about how the environment influences TEs in camelids. Such questions cannot be addressed in the absence of a high quality TE annotation and our work aims to bridge this gap.

In this work, we use a variety of bioinformatics approaches to identify and classify camelid TEs, following the system used by [103]. This system defines two classes of elements according to their transposition mechanism. Class I elements, known as retrotransposable elements (REs), can transpose themselves via an RNA intermediate, self-replicating in the process. REs are the most abundant repetitive elements in many genomes, often including many long terminal repeat REs (LTR-RTs). Class II elements, also known as DNA transposons, can move by means of a "rolling circle" Helitron, or “cut-and-paste” action characterized by terminal inverted repeats (TIRs) of variable length. Within these classes, TEs are further categorized into superfamilies based on homology or structural characteristics.

The great diversity of TEs can make their accurate detection and annotation difficult [61]. Several computational approaches have been developed for identifying TEs in assembled genomes, of which the two main strategies are homology-based and structure-based methods [12]. Additionally, TEs can be uncovered based on their repetitive nature, with queries on the structural signatures of specific TE types supporting the detection of specific types of individual full-length elements; this benefits the investigations of TE variation and evolution [102]. Combining approaches leads to increased sensitivity of detection, resulting in more comprehensive results [71, 74]. In this study, we annotated the TE fraction of the whole genome of each sequenced camelid. We present a detailed approach for the characterization of TEs in camel genomes using homology and structure-based methods, with the aim of providing a basis for future studies on TEs that expand our understanding of genomic diversity and evolution in camelid species. These sequences could be valuable tools for elucidating new genomic dynamics and making evolutionary inferences.

## Methods

### Data source

Four camelid species currently have draft genome sequences available from the National Center of Biotechnology Information (NCBI), assembled at the scaffold level. These sequences were downloaded from the NCBI RefSeq database [79] in FASTA format: *C. dromedarius*, accession GCF_000767585.1, assembled genome size ∼2004 Mb; *C. bactrianus*, GCF_000767855.1, ∼1992 Mb; *C. ferus*, GCF_000311805.1, ∼2009 Mb; and *V. pacos*, GCF_000164845.2, ∼2172 Mb [8, 104]. The genome completeness of the four camelid species was evaluated by Benchmarking Universal Single-Copy Orthologs (BUSCO) v3.0.2 [88], based on mammalian orthologous gene set (4,104 genes).

### Identification of transposable elements

**Fig. 1:**
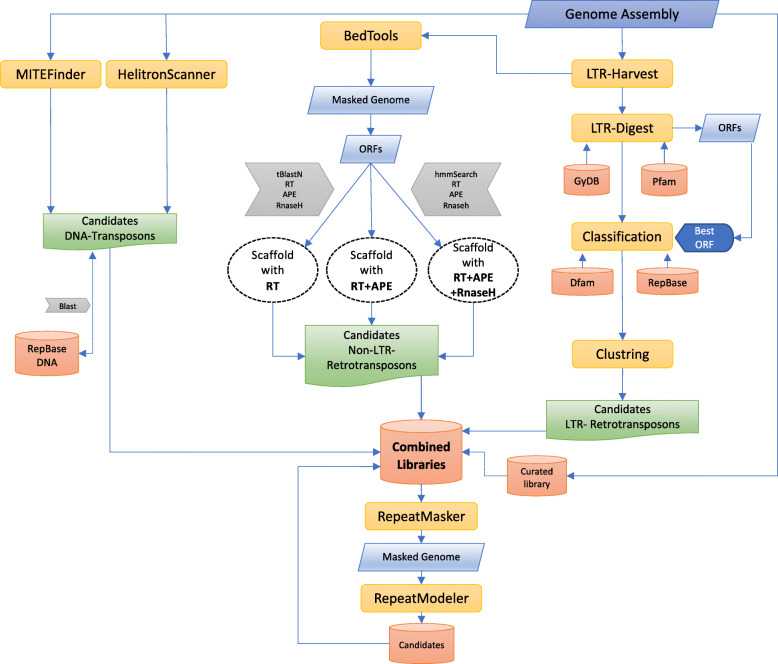
Flowchart for de novo identification of canonical TE sequences using both structural and homology-based approaches

To construct reliable and comprehensive repeat libraries is a challenging task due to the variation in repeat structure and the difficulty of assembling repeats in genome sequences. As many elements vary considerably in genetic structure and sequence, the only means of achieving reliable results when identifying and annotating TEs is to practice complementary approaches [80]. A flowchart describing our overall approach to TE identification is given in Fig. 1. The specific methods for each type are detailed below. We employ de novo signature-based detection programs that rely upon prior knowledge concerning the sharing between different TEs of standard architectural features necessary for the process of transposition. Examples of classification according to similarity to known TEs include records in databases like Repbase [51] and protein profiles retrieved from the Pfam database [31]. Unfortunately, only well-described TEs that have a robust structural signature can be discovered by these methods. Some TEs do not have such characteristics and thus cannot be distinguished by this approach. In contrast to homology-based methods, signature-based methods are less biased by similarity to the set of known elements.

#### Class I elements: LTR retrotransposons

Candidate LTR-RT loci were identified by employing the program LTRharvest [29], a component of GenomeTools [37], which searches the input sequence for direct repeats (LTRs) that are separated by a given distance (default 1 kb) and outside of which are apparent target site duplications (TSDs). Candidates distinguished by LTRharvest were then passed to LTRdigest [92], which annotates protein-coding domains between the LTRs of each putative element. Specifically, LTRdigest searches for homologs in the putative LTR-RTs using HMMER3 [101] and a set of TE-related pHMMs we provided from Pfam and GyDB [31, 63]. Subsequently, the EMBOSS (v.6.6.0) program getorf [84] was employed to annotate additional ORFs of at least 100 amino acids in length that do not overlap the LTRdigest predictions. Afterwards, the predicted LTR-RTs were investigated for homology to LTR-RT sequences in Dfam and Repbase through applying nhmmer and tblastx, respectively. Elements without homologs were discarded as false positives. Each retained element was considered a true positive and classified according to the superfamily of the highest-scoring entry from Dfam or Repbase. Finally, the elements were clustered using the sequence similarity method suggested by [103], based on the “80-80-80” rule, which allocates at least 80% sequence similarity in ≥80% of the element length with a minimum of 80 bp of aligned segments.

Elements were aligned using MAFFT v7.453 [57], and putative intra-element gene conversion tracts between LTRs were identified in those alignments using GENECONV v.1.81a [86], which detects stretches of greater-than-likely similarity. Putative gene conversion tracts with fewer than three total differences were not accepted. Subsequent analyses were performed on each cluster independently. Relative insertion times were calculated for each element per the method of [85]. First, the LTRs within a cluster were aligned and divergences were estimated using PAUP* [94] under the HKY85 model of nucleotide sequence evolution [42]. Since gene conversion events erase the signal of time in a LTR alignment, divergence estimates were improved under the assumption that the part(s) of an LTR alignment containing a putative gene conversion tract will exhibit the same rate of divergence as the portion of the alignment not participating in a gene conversion tract. Following the approach of [21] for calculating divergence among protein-coding genes, divergence estimates were scaled linearly relative to putative gene conversion tracts. Divergences were converted to millions of years using a previously published estimate of the *C. dromedarius* generational substitution rate of 2.5 x 10^-8^ and a generation time of five years [32]. Phylogenies were inferred for each cluster in order to investigate the evolutionary history of identified elements. Whole elements were aligned by MAFFT using 2 or three iterations of the FFT-NS-2 algorithm respectively for clusters with 50 and fewer elements or between 50 and 500 elements, and a single iteration of the FFT-NS-1 algorithm for clusters having between 500 and 1000 elements. Alignments were post-processed with trimAl [18] using the setting -automated1, and evolutionary trees were inferred using FastTree2 [77] under the GTR-CAT model. Phylogenies and LTR-RT diagrams were visualized using FigTree v1.4.4 (2018) and ETE3 [48]. We also employed Kruskal-Wallis and t-test using R stats package (R Core Team, 2019) to test for differences in underlying LTR-RT length distributions and means between species, respectively.

#### Class I elements: Non-LTR retrotransposons

Here, we began with the recognized genomic coordinates of LTR-RTs identified in the previous step. These candidates were masked with maskfasta from BedTools [81] to avoid conflicts or duplicate hits. Next, open reading frame sequences were extracted from the masked genome by applying the getorf tool from EMBOSS v6.4.0.0. The minimum ORF size was set to 500 bp in anticipation of detecting the apyrimidinic endonuclease (APE) gene (which is 600–800 bp in 97% of inspected non-LTR elements). Non-LTRs have been previously classified into clades or lineages [65], and subsequently into families. In only two clades, the reverse transcriptase (RT) is encoded by a single domain (R2 and CRE clades). The others have an additional coding region for an APE. Some elements, such as those belonging to clade I, have an extra RNaseH domain [106]. Accordingly, we performed an exploration of the genomic sequences with MGEScan-non-LTR [83], which identifies and classifies non-LTR TEs in genomic sequences using probabilistic models based on the structure of the 12 established non-LTR TE clades. More precisely, we used MGEScan-non-LTR and hmmsearch from HMMER 3.0 [28] with two separate hidden Markov model (HMM) profiles, one for the reverse transcriptase (RT) gene and one for the endonuclease (APE) gene, both of which are well conserved among non-LTR TEs.

#### Class II elements

All eukaryotic DNA transposons reported so far belong to a single category of elements which use the so-called “cut-and-paste” transposition mechanism, except Helitrons, which transpose by rolling-circle replication. Here, we employed methodologies for the detection of DNA transposons in the studied genomes based on the initial identification of TIR, and non-autonomous elements such as miniature inverted-repeat elements (MITEs) and helitron.

MITEs are DNA-based elements that have TIRs but lack a transposase gene, and their well-defined structural features make them suitable for discovery by computational approaches. We utilized an accurate, valuable tool for detecting MITEs in eukaryotic genomes, MiteFinderII [45]; this tool is capable of detecting both perfect and imperfect inverted repeats through a string matching approach [108]. It computes a new function to cluster MITE sequences into different MITE families in several steps. First, it builds a k-mer index and seeks inverted repeats. Then, all sequence candidates are distinguished by the presence of a TIR pair of default length and a TSD pair. Second, the scaffolds are divided into multiple sequence fragments that overlap by 800 bp, which is the maximum length of MITEs, to guarantee that all inverted repeats are identified. Third, pairs of TIRs having lengths in the range of 50-800 bp are retained, and the remainder used as seeds for MITE candidates in the next step. Finally, identified sequences are compared with MITEs in the Repbase database using blastn [5]. Those with high similarity are considered valid positives, and those with low similarity as false positives. For each MITE cluster, the sequence with the highest blast score was selected via an in-house script as the representative family sequence. The tool was executed with default parameters, except for the use of a confidence-score threshold of 0.5 to exclude low-confidence candidates.

Helitrons are diverse across species and even within one species. These are rolling circle eukaryotic transposons that regularly catch gene sequences and do not form target site duplications or end in TIRs. To investigate the presence of Helitrons in camelid genome, we used the structure-based tool HelitronScanner [105]. HelitronScanner relies on sequence matches between trained local combinational variables (LCVs) and genome. Specifically, it scores 5 ^′^ and 3 ^′^ termini based on a training set of published Helitrons, and then merges the coordinates and scores for putative Helitron-like sequences. We increased the threshold to 6 to avoid false positives. The predicted candidates were clustered using CD-Hit [34] at 80% similarity. Finally, we used tBlastn against Helitron sequences deposited in RepBase-20181026 to retrieve the highest scores.

#### Transposable element annotation, copy number and genome coverage estimation

After all libraries were generated using the programs mentioned above, the TE repeat sequences present in Camelidae species were extracted from Dfam Consensus-20170127 and RepBase-20181026 using the script “queryRepeatDatabase.pl” shipped with RepeatMasker. The results of both steps above were combined. Next, duplicates were filtered using seqKit rmdup (-s) on the basis of sequence. We then used RepeatMasker v.4.1.0 [90] to process the results for masking and annotation [87]. We used RMBlast as the search algorithm with a Smith-Waterman cutoff of 225, -no_is, -gff -s -lib, -norna and exclusion of low complexity regions -nolow; all other parameters were default. Additionally, counting the copy number of each TE and determined genome coverage obtained from the RepeatMasker output files (.out), which correspond to the number of insertions identified in the masked genomes. The remaining unmasked portion of the genome is scanned using RepeatModeler [33] with default settings to detect any unclassified TEs such as TIRs that were missed by structure-based TE identification and merge it to the library for Re-annotation.

## Results

### Evaluation of genome assemblies

**Fig. 2:**
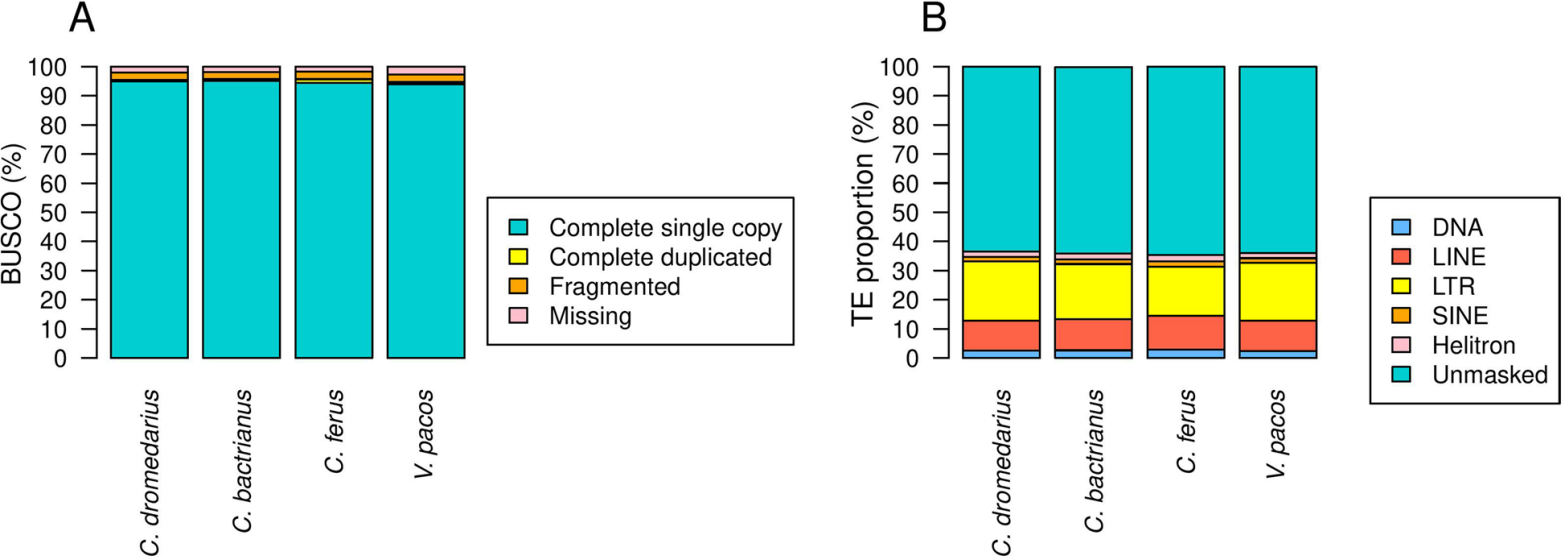
Genome assembly assessment and TE proportions. The BUSCO dataset of the mammalia_db9 including 4,104 BUSCO was utilized to evaluate the four camelid assemblies (**A**). TE proportions in the four camelid genomes (**B**)

To evaluate the completeness of each of the four camelid genome assemblies, we used BUSCO mammalian lineage dataset (mammalia_odb9), which consisted of 4,104 single-copy orthologs. BUSCO results showed that 93.9–95.2% of the 4104 mammalian single-copy orthologs were complete across the four genome assemblies (Fig. 2A), suggesting the four genomes are comparable and have high-quality assemblies.

### Construction of camelid repeat libraries

TE reference libraries were generated through systematic searching procedures using both de novo signature-based and homology-based method strategies for four camelid draft genomes: *C. dromedarius*, *C. bactrianus*, *C. ferus*, and *V. pacos*. The joined libraries include related repeats deposited in Dfam and Repbase. They respectively consist of a total of 6026, 6594, 7241, and 8311 individual TE sequences, and cover both Class I (LTR-RTs, non-LTR retrotransposons) and Class II elements (TIR elements, Helitrons, and MITEs) 41,701,493 bp, 32,935,780 bp, 26,533,293 bp, and 39,127,481 bp respectively (Supplementary files S1–S4). The TE sequence annotations generated by these libraries respectively comprise 734,069,793 bp (36.63%), 718,445,875 bp (36.05%), 711,915,199 bp (35.43%), and 783,294,957 bp (36.06%) of the investigated camel genomes (Table 1, Fig. 2B, and Supplementary files S5–S8).

**Table 1:** Summary of transposable elements identified in camelid draft genomes using a species-specific de novo library

		*C. dromedarius*			*C. bactrianus*			*C. ferus*			*V. pacos*		
Type	Subtype	Number	Length (bp)	%	Number	Length (bp)	%	Number	Length (bp)	%	Number	Length (bp)	%
SINEs		278868	31857598	1.59	281475	33148009	1.66	283439	35670873	1.78	283725	33811705	1.56
	Alu/B1	0	0	0.00	0	0	0.00	0	0	0.00	0	0	0.00
	MIRs	277345	31646134	1.58	279944	32933495	1.65	281911	35456137	1.76	282207	33598228	1.55
LINEs		529041	205166126	10.24	533744	212014107	10.64	549855	231412043	11.52	581214	226910732	10.45
	LINE1	350498	156418155	7.81	354589	162643009	8.16	366661	181254065	9.02	396675	176469418	8.12
	LINE2	148769	41148875	2.05	149561	41824413	2.10	153057	42340613	2.11	154447	42875454	1.97
	L3/CR1	18721	4530167	0.23	18851	4558065	0.23	18962	4611963	0.23	19141	4639455	0.21
	RTE	9419	2660649	0.13	9360	2664989	0.13	9435	2693791	0.13	9562	2642926	0.12
LTR elements		2594909	407821551	20.35	2419041	378419511	18.99	2331615	339335142	16.89	2665421	431052951	19.84
	ERVL	662080	105706311	5.27	605948	101811660	5.11	533741	83612548	4.16	654184	107467377	4.95
	ERVL-MaLRs	1026977	162691546	8.12	849467	141960364	7.12	777378	116645292	5.81	1031590	165237845	7.61
	ERV_classI	475024	80237999	4.00	561059	87160641	4.37	574643	85619681	4.26	529102	94406478	4.35
	ERV_classII	210298	24634450	1.23	181906	19392527	0.97	186502	23168720	1.15	232541	27934704	1.29
DNA transposons		276980	50575476	2.52	280059	52067181	2.61	284093	58877931	2.93	285372	52393874	2.41
	hAT-Charlie	162951	25352221	1.27	164860	26366817	1.32	166487	31267569	1.56	167370	26234946	1.21
	TcMar-Tigger	41555	11263825	0.56	42108	11407670	0.57	43542	12529407	0.62	43728	11791963	0.54
Helitrons		386049	38090118	1.90	443857	39651774	1.99	487720	46043445	2.29	379112	37564847	1.73
Unclassified		3508	564776	0.03	3516	568373	0.03	3556	574586	0.03	3538	565582	0.03
Total		4069355	734069793	36.63	3961692	718445875	36.05	3940278	711915199	35.43	4198382	783294957	36.06

Our findings revealed the relative contributions to camelid genome of significant types of TEs, namely LTR, LINE, and SINE retrotransposons, as well as DNA transposons, (Fig. 2B). Here, we employed several procedures to identify each order of TEs present in four camelid genomes: *C. dromedarius*, *C. bactrianus*, *C. ferus*, and *V. pacos*. The results are classified into four main categories: 1) LTR-RTs elements identified using LTRharvest, and internal regions annotated employing LTRdigest: these comprised respective totals of 4473, 4794, 5768, and 6877 elements in the studied genomes, and open reading frames (ORFs) were simultaneously distinguished. 2) Non-LTR retrotransposons were identified by aligning reverse transcriptase accessions to ORFs predicted in the genomes, yielding 495, 475, 87, and 11 sequences, respectively. 3) Non-autonomous DNA elements (MITEs and degraded DNA transposons) were identified by their TIRs and TSDs using MITE-FinderII, yielding 96, 76, 73, and 64 families respectively. 4) Helitron-like sequences were identified using HelitronScanner, and consisted of 532, 557, 503, and 524 sequences, respectively.

#### LTR retrotransposons

LTR retrotransposons appear to dominate the camel genomes, being the most significant component among the identified TEs (Fig. 2B).

In the *C. dromedarius* genome, LTRharvest identified 11303 candidate LTR-RTs each consisting of two relatively intact LTRs and flanking TSDs. After LTRdigest annotation analyses, discarding the false-positive candidates reduced the number to 4473 putative full-length LTR-RTs, which comprise 39% of the total predicted candidates. The lengths of these elements range from 205 to 25,500 bp, with an average of 7,691.2 bp and a total genome-wide footprint of 34,402,528 bp.

In the *C. bactrianus* genome, LTRharvest predicted 10920 candidate LTR-RTs with two relatively intact LTRs and flanking TSDs. After discarding false positives, reduced the number to 4,794 putative full-length LTR-RTs, comprising 43% of the total predicted candidates. The lengths of these elements ranged from 203 to 15,702 bp, with an average size of 5,211 bp and a total footprint of 24,981,380 bp.

In the *C. ferus* genome, LTRharvest identified 17456 LTR-RTs candidates with two relatively intact LTRs and flanking TSDs. Discarding false-positive candidates reduced the list to 5768 putative full-length LTR-RTs, comprised 33% of the total predicted candidates. Their lengths ranged from 203 to 15,701 bp, with an average size of 3,480.4 bp and a total footprint of 20,075,064 bp.

In the *V. pacos* genome, LTRharvest predicted 24674 candidate LTR-RTs with two relatively intact LTRs and flanking TSDs. Removal of false positives reduced the total to 6877 putative full-length LTR-RTs, comprised 27% of all predicted candidates. These elements ranged from 208 to 15,887 bp in length, with an average of 4,757.2 bp and a total footprint of 32,715,308 bp.

The identified LTR-RTs were classified into 11 superfamilies according to similarity (Table 2). In all four genomes, the most abundant LTR-RT superfamily was ERVL-MaLR (1503-2031 elements). The second most prevalent was either ERV1 (1142–2455) or ERVL (987–1316), followed by ERV2 (410–679) and then Gypsy (15–56). Similar numbers were found in each genome for the ERVL, ERV1, and ERV2 families, but counts of ERV1, ERVL, and ERVL-MaLR elements were greater in genomes with successively more total LTR-RTs (Table 3).

**Table 2:** De novo classification of predicted Class I LTR retrotransposons into superfamilies based on homology to labeled sequences in Dfam and Repbase

Classification	*C. dromedarius*	*C. bactrianus*	*C. ferus*	*V. pacos*
ERVL	987	1139	1297	1316
ERVL-MaLR	1503	1708	1881	2031
ERV1	1142	1160	1584	2455
ERV2	439	410	561	679
Gypsy	32	15	42	56
Copia	84	78	119	60
DIRS	117	120	46	56
Ngaro	15	14	45	25
Pao	6	2	8	4
Unknown	29	29	32	42
Undefined	119	119	153	153
Total	4,473	4,794	5,768	6,877

**Table 3:** Counts of LTR-RTs with evidence of intra-element gene conversion

Species	Elements with gene conversion
*C. dromedarius*	1,886
*C. bactrianus*	1,765
*C. ferus*	1,908
*V. pacos*	1,787

**Fig. 3:**
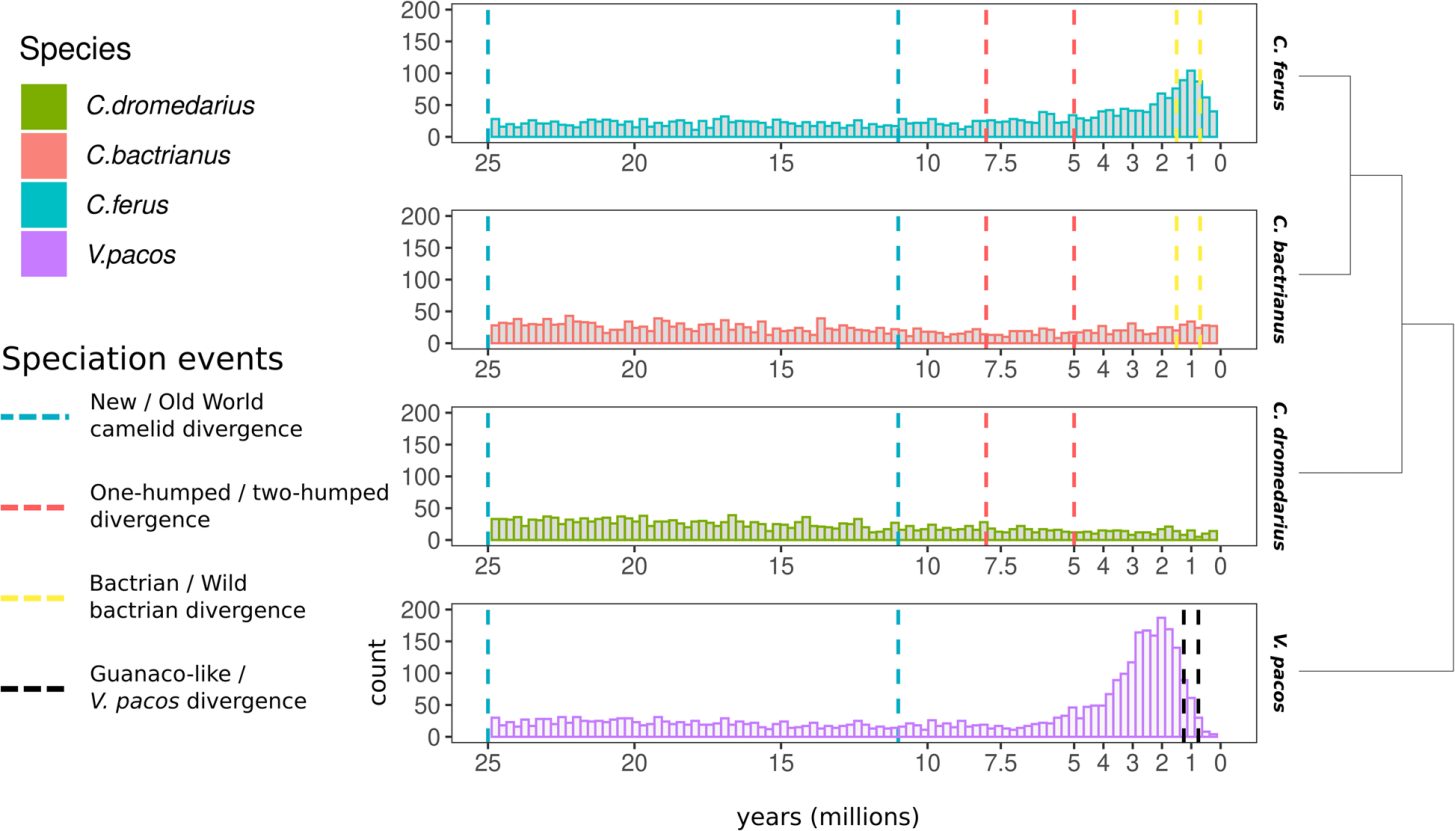
Distributions of LTR-RT ages in each of the four camelid genomes, with speciation event interval demarcated by vertical dashed lines. Phylogenetic relationships among species are depicted by the tree on the right

**Fig. 4:**
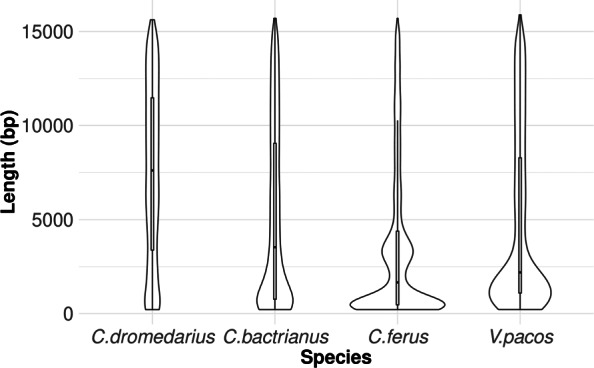
LTR-RT length distributions in the four camelid genomes

Histograms of intra-element ages by-species are depicted in Fig. 3. When divergences were scaled based on putative gene conversion tracts, the distribution shapes remained very similar to those of the unaltered divergences, except for having long tails; therefore, the unaltered divergences are shown. In the genomes of *C. dromedarius* and *C. bactrianus*, LTR-RTs appear to have had consistently relatively low activity for the past 25 million years (mya). This contrasts with LTR-RTs in *C. ferus* and *V. pacos*, where there have been recent bursts between about 0.5 - 2.5 mya and 1.5 - 4 mya, respectively (Fig. 3). Notably, the distributions of LTR-RT lengths differed among species (Fig. 4, Tables 4 and 5). Normalized to *C. dromedarius*, the mean LTR-RT lengths of the other three studied species were 0.69 (*C. bactrianus*), 0.46 (*C. ferus*), and 0.63 (*V. pacos*).

**Table 4:** Results of Kruskal-Wallis test for LTR-RT length distributions between species. Significance (p <= 0.05) indicates the LTR-RT lengths follow a different distribution in the two species

	*C. dromedarius*	*C. bactrianus*	*C. ferus*	*V. pacos*
*C. dromedarius*	1	2.8E-189	1.4E-118	0
*C. bactrianus*	2.8E-189	1	0.016	4.6E-88
*C. ferus*	1.4E-118	0.016	1	3.7E-84
*V. pacos*	0	4.6E-88	3.7E-84	1

**Table 5:** Results of t-test test for differences in LTR-RT length means. Significance (*p* <= 0.05) indicates the mean LTR-RT length differs between the two species

	*C. dromedarius*	*C. bactrianus*	*C. ferus*	*V. pacos*
*C. dromedarius*	1	5.4E-204	6.1E-124	0
*C. bactrianus*	5.4E-204	1	7.9E-7	9.2E-67
*C. ferus*	6.1E-124	7.9E-7	1	1.8E-91
*V. pacos*	0	9.2E-67	1.8E-91	1

**Fig. 5:**
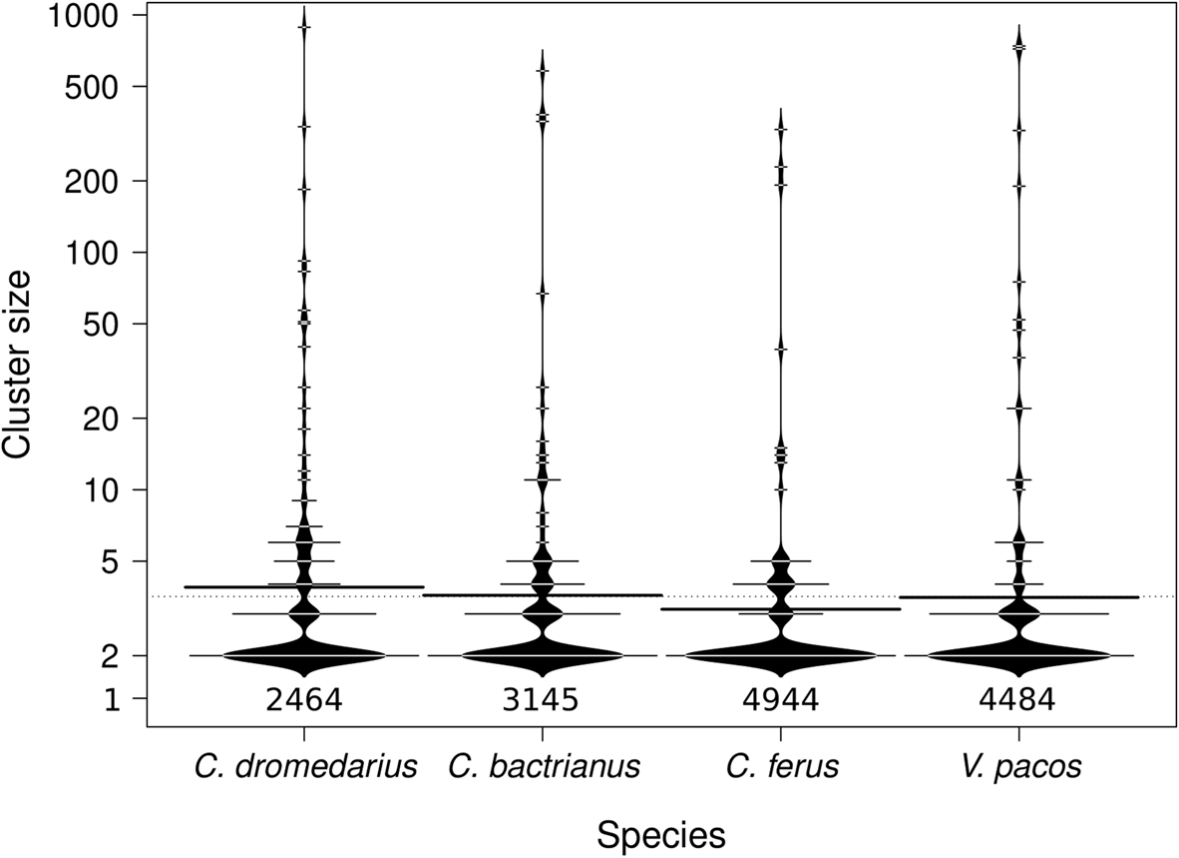
Distributions of LTR-RT cluster sizes for clusters delineated using the 80-80-80 rule. The y-axis is log(10)-transformed. Small horizontal lines represent individual clusters; their lengths are proportional to the number of clusters with that particular size, with the exception of the line for cluster size = 2, which is shortened to save space. Thick horizontal lines are means and the dotted horizontal line is the overall mean. Singletons are included as counts at the bottom of the plot

**Fig. 6:**
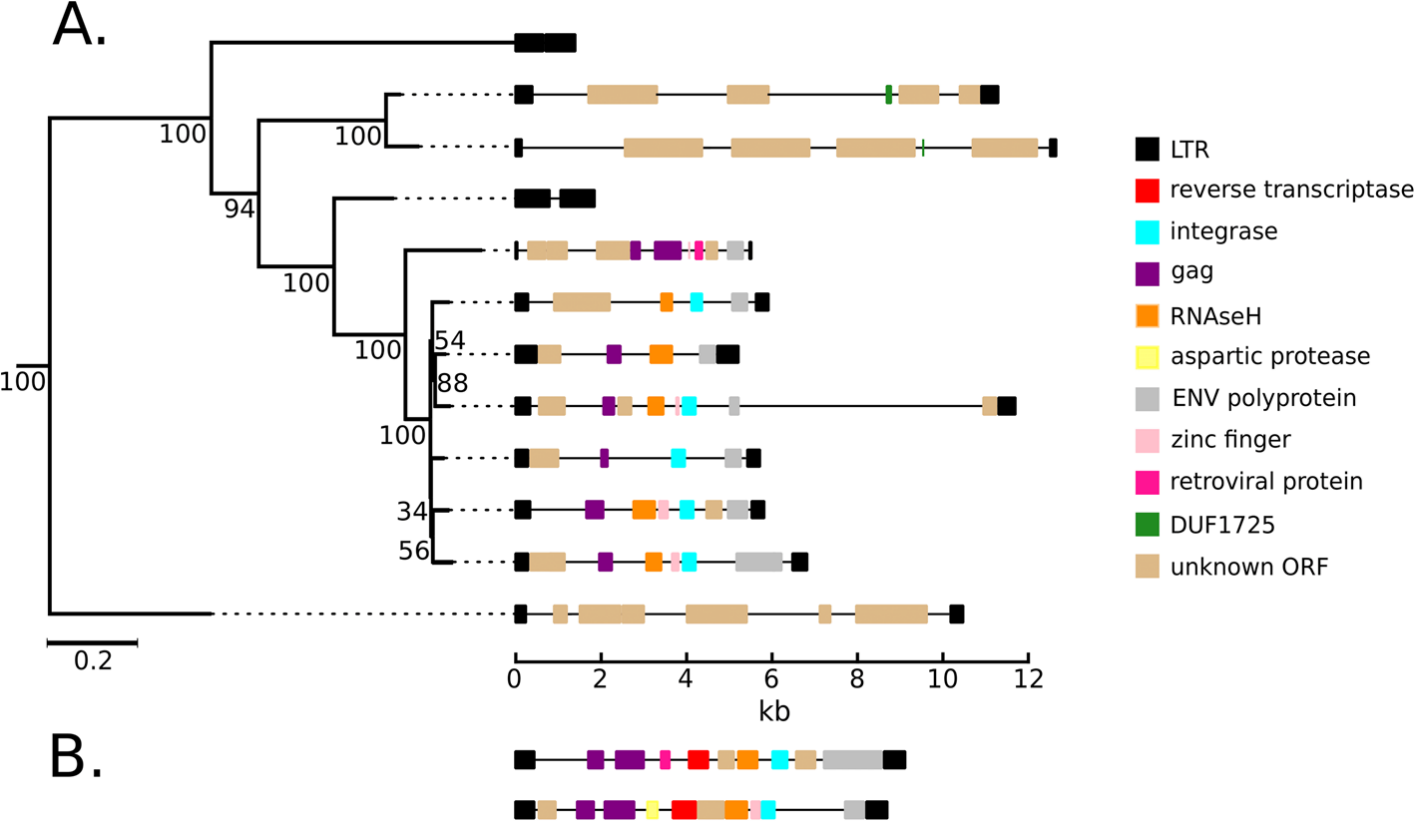
Phylogenetic tree for LTR-RTs in cluster 6 of the ERV1 group in *Vicugna pacos*, with diagrams showing LTR domain structure (**A**) and two exemplar elements from cluster 4 (**B**)

**Fig. 7:**
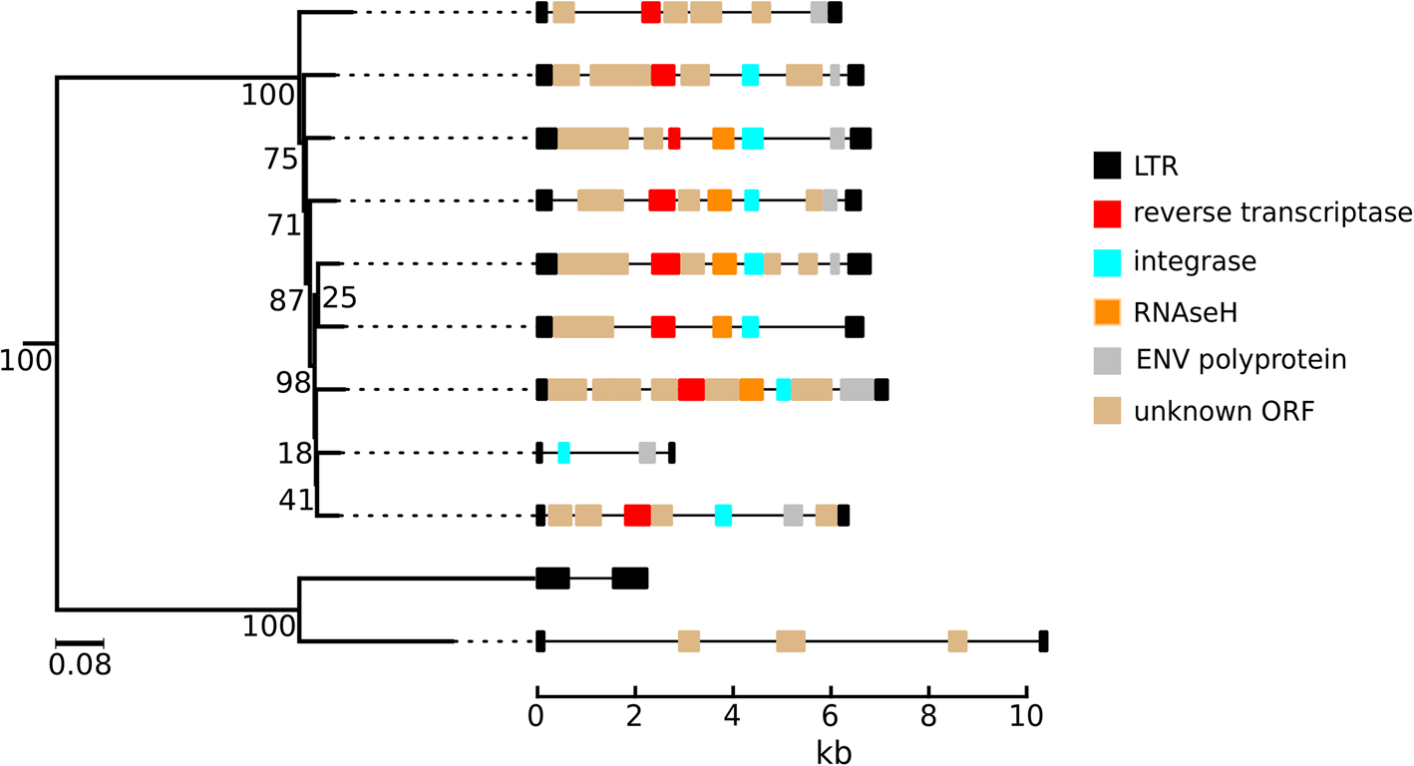
Phylogenetic tree for LTR-RTs in cluster 2 of the ERVL group in *V. pacos*, with diagrams showing LTR domain structure

Bean-plots of cluster size distributions show similar patterns for all of the species, predominantly consisting of singletons and smaller clusters (Fig. 5). The vast majority of clusters contain elements that are heterogeneous in length; relatively few contained the suite of domains necessary for transposition, as recognizable through high similarity to entries from Pfam and GyDB. However, there are generally multiple ORFs in each element; it is likely that at least some of these encode transposition machinery, but are too divergent to be detected by the pHMM search. The phylogenies of most clusters have poor bootstrap support, with the exceptions of three small clusters in the *V. pacos* genome, two of which ERV1 clusters (Fig. 6) and one ERVL (Fig. 7). The ERV1 LTR-RTs in *V. pacos* are also remarkable for the presence of multiple large clusters that almost exclusively contain short elements, most of which have no internal ORFs or identifiable LTR-RT-related protein-coding domains. These elements may be non-autonomous and rely on protein products derived from other elements.

The protein sequences identified internal to LTR-RTs are summarized in Table 6. In total, 2889, 2350, 1365, and 2409 proteins were respectively identified in the four species (*C. dromedarius*, *C. bactrianus*, *C. ferus*, and *V. pacos*) (Table 6). Most of the putative LTR-RTs contained the RT-INT-ENV protein domain order (reverse transcriptase, integrase, and envelop) characteristic of ERVs.

**Table 6:** Summary of all protein hits detected in the four camelid genomes

Classification	*C. dromedarius*	*C. bactrianus*	*C. ferus*	*V. pacos*
Gag_p10	13	5	0	19
Gag_p24	16	4	1	19
Gag_p30	95	56	23	95
gag-asp_proteas	4	2	1	5
Gag_MA	57	35	11	63
Asp	1	0	0	0
Asp_protease_2	2	1	1	3
HTH_Tnp_Tc5	2	2	3	3
IN_DBD_C	9	6	4	8
zf_C2H2	12	24	29	12
zf_CCHC	25	14	7	21
zf_CCHC_5	12	4	2	15
zf_H2C2	15	8	5	14
zf_H3C2	0	0	0	1
zfCCHC_2	2	1	0	0
zfCCHC_3	0	0	0	1
Integrase_Zn	14	4	4	17
dUTPase	22	9	5	19
Exo_endo_phos_2	60	39	5	10
RNase_H	67	37	14	70
Transposase_22	247	262	126	165
N-Term_TEN	0	0	4	0
RVP	46	25	11	45
RVT_thumb	23	10	5	18
TLV_coat	95	63	27	109
rve	95	47	26	96
rve_3	0	0	1	0
RVT_1	511	395	184	358
RVT_2	1	0	0	0
DUF1725	1443	1299	863	1228
Total	2889	2350	1365	2409

#### Non-LTR retrotransposons

Non-LTR retrotransposons were identified by applying MGEScan-non-LTR to the LTR-masked genomes. This tool discovered all known full-length elements and simultaneously classified them into the following clades: CRE, I, Jockey, L1, R1, R2, and RTE. Notably, reverse transcriptase (RT) is encoded by all autonomous non-LTR retrotransposons, and therefore was used as the primary signal to distinguish and classify these elements. Previous studies have classified non-LTR retrotransposons into 11 clades based on RT phylogeny [65].

The non-LTR retrotransposons identified in camelid species are summarized in Table 7. Six clades were represented in *C. dromedarius*, five in *C. bactrianus*, four in *C. ferus*, and six in *V. pacos*. In the three *Camelus* species, L1 was the most abundant clade, represented by 442, 421, and 41 elements and comprising total footprints of 1,003,622 bp, 1,571,496 bp, and 490,265 bp in *C. dromedarius*, *C. bactrianus*, and *C. ferus*, respectively. Surprisingly, the smallest number of ORF-conserving elements was identified in *V. pacos*; these elements collectively occupied 13,785 bp.

**Table 7:** Counts of ORF-preserving non-LTR retrotransposons identified in the four camelid genomes

Clade	*C. dromedarius*	*C. bactrianus*	*C. ferus*	*V. pacos*
CR1	0	1	0	0
CRE	0	0	0	1
I	20	18	9	2
Jockey	27	31	18	1
L1	442	424	59	1
L2	-	-	-	-
R1	2	0	0	0
R2	3	1	1	5
RandI	-	-	-	-
Rex	-	-	-	-
RTE	1	0	0	1
Tad1	-	-	-	-
Total	495	475	87	11

#### DNA transposons

Miniature inverted-repeat elements (MITEs) were one such ubiquitous class, characterized by essential structural features such as TIRs and TSDs, AT-rich sequences, and a lack of coding capacity for transposases. Canonical MITE sequences with perfect TSDs, perfect or near-perfect TIR structure, and a length was between 50 and 650 bp were counted using MITEFinder software; a total of 285 families across the four examined genomes. Relative empty site analysis showed that the TSD sequences differed between families, being either 2, 3, 8, or 10 bp. Moreover, we performed homology-based repeat analysis on the library of identified camelid MITEs using a subsection of the Repbase database carrying only class II vertebrate and mammalian sequences. The resulting superfamily classifications and the number of families detected in each species are given in Table 8.

**Table 8:** De novo classification of predicted Class II MITEs into superfamilies based on homology via altered Repbase

Classification	*C. dromedarius*	*C. bactrianus*	*C. ferus*	*V. pacos*
haT	29	29	27	26
CACTA/EnSpm	12	16	15	16
Tc1-Mariner	6	14	11	4
Harbinger	5	6	7	5
piggyBac	4	4	4	4
Merlin	4	3	3	1
Kolobok	6	3	3	3
P	1	0	2	2
Mud	2	2	1	3
Helitron	532	557	503	524
Total	601	661	576	568

In the *C. dromedarius* genome, we identified a total of 69 MITE elements, which accounted for 13,922 bp of the genome and comprised 9 different families. In *C. bactrianus*, we identified 76 MITE elements, which accounted for 14,364 bp of the genome and clustered into 7 families, 74 of them being present in Repbase (1997 hits). In *C. ferus*, we identified 73 MITE elements, which account for 14,092 bp of the genome and clustered into 9 families, 72 of them being present in Repbase. Finally, in the *V. pacos* genome, 64 MITE elements were identified, which accounted for 12,087 bp of the genome and clustered into 9 families, 62 of them being present in Repbase (1908 hits). In all four species evaluated, the most abundant superfamily of DNA transposons was hAT, represented by 26-29 families.

Finally, we employed HelitronScanner to identify Helitron-like sequences using a structure-based approach. HelitronScanner aims to extract more definitive Helitron features than the few previously identified: the TC dinucleotide at the 5 ^′^ end, the hairpin structure, the CTRR (R = A or G) sequence at the 3 ^′^ end, and the A and T residues respectively flanking the 5 ^′^ and 3 ^′^ ends. It assigns to each identified Helitron a LCV score, which is an indicator of prediction confidence; we considered elements with scores of 6 or greater for each end. More than 500 double-ended Helitron sequences were identified in each camel genome (Table 8). These respectively accounted for 5,702,516 bp, 5,774,487 bp, 5,355,741 bp, and 5,491,739 bp total in *C. dromedarius*, *C. bactrianus*, *C. ferus*, and *V. pacos*. These candidates were classified into superfamilies based on homology (Table 9), with the most abundant being Helitron DR (225, 237, 204, and 223 total elements) and Helitron GA (31, 32, 29, and 26 total elements).

**Table 9:** The best predicted classification returned of Class II Helitron candidates into superfamilies based on homology via altered Repbase

	*C. dromedarius*	*C. bactrianus*	*C. ferus*	*V. pacos*
Helitron-2_DR	62	53	60	64
Helitron-4_DR	54	67	57	67
Helitron-1_DR	49	50	43	37
Helitron-5_DR	26	31	18	25
Helitron-N3_DR	19	21	15	18
Helitron-N3b_DR	15	15	11	12
Helitron-1_GA	31	32	29	26
Helitron-1_OL	29	37	31	23
Helitron-1_AC	16	16	17	23
Helitron-N3_EL	14	16	15	8

## Discussion

We generated repeat libraries for each of the four camel species with available genome sequences in order to investigate the abundance and character of repeat-derived DNA within their genomes, as well as to facilitate the repeat-masking of DNA in future studies. Notably, we worked on assembled genome drafts, which frequently do not include TE-rich regions like centromeres or other heterochromatic regions. Our analysis techniques were also very conservative and may have dropped other types of TEs or elements that are ancient and divergent. To ensure the reliability of our results, we employed a method incorporating both known TEs and signature-based repeat identification tools.

TEs are abundant in almost all living organisms and closely related species have similar TE content [13, 40]. Here, our repeat analysis reveals significant similarity in total TE content (35.43–36.63%) between genome assemblies of the four species (Fig. 2B), which were consistent with lizards (34.4%) [3], carp (31.3%) [107], and western clawed frog (34.5%) [43]. Interestingly, early studies on camelid cytogenetics have evidenced a striking uniformity in the karyotypes of Asian and South American camelids, despite significant divergence times and adaptation to different environments [10, 16, 95]. In this sense, our result of similarity in the overall repeat content between camelid genomes could reflect a general evolutionary trend of genome stability in this family.

Overall, our investigation found that camelid genomes are characterized by a strong predominance of retroelements over DNA transposons. However, the genomes were similar in the relative abundances of element types (LTRs > non-LTRs > DNA) (Fig. 2B). Class I elements constituted 22–32% of the annotated genomes, with LTRs comprising 16–20%, SINEs only about 2%, and LINEs 10–11%. The observed high abundance of LTR might be a distinctive feature of camelids. However, this pattern is typical for plants compared to other mammals and vertebrates [68, 73, 93], and this could reflect the high variability of TE abundance across vertebrate genomes [22, 75]. Class II elements collectively made up 4–5% of the annotated genomes, with DNA Transposons comprising about 2–3% and Helitrons about 2%. Notably, DNA repeat proliferation is one of the key factors that influence species differences in genome size and composition; others include whole-genome duplications, segmental duplications, and deletions [72]. Our findings revealed that the examined camel genomes have similar total repetitive makeup, with unique genome content comprising 1.26, 1.27, 1.29, and 1.38 GB respectively in *C. dromedarius*, *C. bactrianus*, *C. ferus*, and *V. pacos*. [30] suggested that larger genomes might have more TEs. The observed differences between genomes could also stem from technical limitations in sequencing and assembling repeats using short-read sequencing.

LTR-RTs are usually more abundant than other types of TEs, and so the identification of full-length elements benefits research into the structural variability, diversity, and phylogenetic evolution of TEs in camelid genomes. Accordingly, we investigated full-length LTR-RTs in camelid genomes in detail. We discovered that the most abundant full-length LTRs were ERVs, including the ERVL, ERVL-MaLR, ERV classI, and ERV classII superfamilies. ERVs are remnants of past retroviral infections, which may have arisen within genomes by at least two different mechanisms: retrotransposition from a pre-existing endogenous retrovirus or the infection and integration of an exogenous source virus [50]. LTR-RTs have also been domesticated numerous times to perform roles in functions such as placental development, host defence against exogenous retroviruses, brain development, and more [70]. Among camelid ERVs, ERV-MaLR was the most frequent; this element is assumed to have been inserted into the mammalian genome about 70 million years ago [11]. Studies have revealed that ERV classII is one of the youngest members of endogenous retroviruses [56], comprising only a low percentage of animal genomes [1]. Consistent with this data, this study, found ERV Class II to have the lowest representation of all ERV elements across all four camel genomes.

To help elucidate the evolutionary history of camelid LTR-RTs, we dated insertions of each full-length LTR-RT by estimating sequence divergence (substitutions per site) between long terminal repeats and scaling them by an estimate of the *C. dromedarius* nuclear genome generational mutation rate [32]. Considering the timing of evolutionarily significant events such as speciations, distributions of LTR-RT abundance across time can suggest mechanisms that may have facilitated evolutionary change. For example, hybrids often face massive TE de-repression due to widespread DNA de-methylation, leading to a surge in transposition activity [60]. In *C. ferus*, there is a peak of LTR-RT abundance between about 0.3–2.1 mya, corresponding to the time of the speciation process that separated the *C. ferus* from the *C. bactrianus* lineage [17]. The *V. pacos* lineage experienced a burst of LTR-RT activity from about 1–4 mya, a range that overlaps the 0.78–1.3 mya interval during which *V. pacos* diverged from the extant or an extinct lineage of guanaco [25] only slightly; during a time LTR-RTs were less active than they were about a million years prior during the peak of the burst. Therefore, the increased transposition rate could reflect a process occurring in the lineage of the common ancestor of *V. pacos* and the extant or wild guanaco. It would be interesting to see a distribution of LTR-RT insertion dates for the exant wild guanaco. If the distribution shares the peak of LTR-RT abundances aged 1–4 mya observed in *V. pacos*, then it would suggest the *V. pacos* lineage diverged from the extant wild guanaco lineage more than 4 mya. If the distribution does not share the peak, it would suggest *V. pacos* is derived from an extinct wild guanaco lineage, in agreement with the model of llama domestication proposed by [26]. It would also be interesting to investigate whether genes in *C. ferus* and *V. pacos* have LTR-RT insertions near promoters of transcription factors or other genes which might be implicated in phenotypic differences between these species [7].

We determined that LINE elements were the most prevalent non-LTR repeat in camelids, contributing 10–11% of the total assembled genomes (Table 1). The LINE proportion in Camelids seems very similar to that of lizards (12.34%) and higher than in birds (6%), coelacanth (6.43%), cod (3.3%), and western clawed frog (5.4%) [3, 43, 98]. Among LINEs, the LINE1 (L1) represents the most abundant family in mammals [59], and here confirmed to be the most abundant in camelids (Table 1). Elements that depend on L1-encoded proteins for retrotransposition are responsible for new germline insertions, mostly in AT-rich regions, that can cause genetic diseases [9]. Moreover, L1 is capable of 3 ^′^ transduction [69]. In contrast, the RTE clade was the least abundant LINE superfamily in camelids. The RTE ORF appears most intimately related to the corresponding ORF of the CR1 autonomous element, another LINE clade which is predominantly found in avian and reptile genomes [66]. A small proportion of camelid genomes (about 0.2%) was found to consist of CR1 elements, but these were determined to have degenerated and become nonfunctional [98, 99].

The contribution of SINEs to camelid genome content was much less significant than that of LINEs, comprising only about 2% of each species’ total genome length (Table 1). SINEs evolved from RNA genes, such as 7SL and tRNA genes [89]. By definition, they are short, measuring up to 1000 base pairs long. They do not encode their retrotransposition machinery and are considered non-autonomous elements. In most cases, SINES are mobilized by L1-derived machinery [53]. Another characteristic of SINEs is that they accept RNA polymerase III transcription [24]. The Alu clade of SINE elements is mostly enriched in GC-rich or gene-rich regions, and considered an abundant and conserved repeat family in primate genomes [39]. This study observed no Alu elements in camel genomes. Instead, we identified mammalian-wide interspersed repeats (MIRs) as the predominant SINE family in camelid genomes, constituting nearly all of the identified SINE elements. MIRs are another prominent SINE clade, whose putative ancestor sequences evolved before the eutherian radiation and spread through mammalian lineages during the Mesozoic era, an estimated 130 million years ago. Accordingly, copies of MIRs have been discovered in diverse mammalian groups, including marsupials and monotremes [52]. [49] suggest that MIRs may play functional roles for their host genomes, and also positively correlate to tissue-specific gene expression. Further research using RNA-seq data could assist us in better understanding the roles of MIRs in camelid genomes.

In this study, we found that DNA transposons constitute about 3% of camelid genomes (Table 1). MITE content has explicitly been estimated in vertebrates, including mammals, birds, frog, and lizard [3, 6, 23, 43, 62, 109]. These elements are usually present in low copy numbers relative to retrotransposons, occupying less than 3% of mammalian genomes [76], consistent with our findings. Previously, scientists believed that the last activity of DNA transposons in mammalian genomes occurred at least 40 million years ago [82]. Their high copy number and structural homogeneity have served to distinguish them from most of the previously described class II elements [100]. We identified a total of 285 DNA transposon families in camelid genomes, which grouped into nine superfamilies based on their TSDs and on known associations in Repbase (Table 8). Of these families, the hAT superfamily predominated in all four studied genomes, and a particular diversity was observed for the hAT and Tc1/Mariner families (Table 1).

Another DNA-based element, Helitrons, are diverse both between species and also within one given species. These elements were first described in plants, but are also present in fungi and animals [44, 78]. They replicate using a rolling-circle mechanism, and their insertion does not result in a TSD [54]. Since their identification, the role of Helitrons in reshaping host genomes has been examined in many organisms, but their actual mechanism of transposition has remained elusive. We employed the tool HelitronScanner [105] to investigate the presence of Helitrons in camelid genomes, and found that they constitute about 2% of the total genome lengths (Table 1). Among the identified camelid Helitrons, the greatest number showed homology to Helitron-2_DR and Helitron-4_DR (Table 9).

In conclusion, the findings of this study will provide a valuable resource for further studies on camel biology. While the present study showed that the investigated genomes had similar contents and distributions of the identified repetitive regions (Fig. 2B), differences were also identified that may be associated with factors such as different evolutionary origins or discrepancies in the assembly stage of these draft genomes. Additional research into camelid repetitive elements, perhaps with more complete genome assemblies, would provide more information about and awareness of the genomic features of camels. Such additional genome-wide detail could improve strategy design for camel maintenance and breeding. Furthermore, the causes and consequences of the high degree of variability that exists in the distribution, amount, and relative proportion of TEs in different genomes are still not wholly understood; it is essential to continue characterizing this critical fraction of eukaryotic genomes. Such characterizations can bring to light evolutionary phenomena, including genomic rearrangements and other dynamic events, that have occurred in the past and may also be under way in contemporary times.

## Data Availability

All data generated or analysed during this study are included in this published article (and its supplementary information files).

## Abbreviations

TEs: Transposable elements
LTR: Long terminal repeat
LINE: Long interspersed element
TIR: Terminal inverted repeat
TSD: Target site duplication
MITEs: Miniature inverted–repeat transposable elements
GyDB: Gypsy database
ORFs: Open reading frames
pHMMs: Profile hidden Markov models
APE: Apurinic/apyrimidinic endonuclease
RNaseH: Ribonuclease H
RT: Reverse transcriptase
